# Global prevalence and factors associated with overweight and obesity in children and adolescents with type 1 diabetes: a systematic review and meta-analysis

**DOI:** 10.1007/s40200-025-01774-7

**Published:** 2025-11-04

**Authors:** Eric Peprah Osei, Emmanuel Ekpor, Gideon Yaw Osei, Samuel Akyirem

**Affiliations:** 1 College of Nursing, University of Illinois Chicago, Chicago, IL USA; 2 School of Nursing and Midwifery, University of Ghana, Accra, Ghana; 3 School of Medical Sciences, Kwame Nkrumah University of Science and Technology, Kumasi, Ghana; 4 Yale School of Nursing, Yale University, New Haven, CT USA

**Keywords:** Children, Obesity, Overweight, Prevalence, Type 1 diabetes

## Abstract

**Objective:**

To determine the global prevalence and factors associated with overweight and obesity in children and adolescents with type 1 diabetes (T1D).

**Methods:**

A systematic database search was conducted in PubMed, CINAHL, EMBASE, MEDLINE, and Web of Science from 2000 to October 2024. Studies were included if they: (1) used observational designs (2) reported the prevalence of overweight and/or obesity among children with TID (< 20 years) (3) were published in English Language. Studies with both T1D and T2D participants that lacked separate T1D findings were excluded. The DerSimonian-Laird random-effects model was used to estimate the pooled prevalence, with heterogeneity assessed using I² statistics. Publication bias was assessed using funnel plots, Egger’s and Begg’s tests.

**Results:**

Out of 10,491 references, 21 articles met the inclusion criteria. The overall pooled prevalence of obesity and overweight among children and adolescents was 30.0%, with substantial geographical and gender differences. The pooled prevalence of obesity alone was 8.8% and overweight prevalence was higher at 20.0%. Key factors such as female gender, increasing age, lower household income, and lower parental education, increased insulin doses, reduced physical activity, lower self-monitoring of blood glucose, perceived stress, poor diabetes care activities, and poor quality of life were associated with higher overweight and obesity risk.

**Conclusion:**

Obesity and overweight in children with T1D stem from a complex interplay of sociodemographic, clinical, and behavioral factors. Data from underrepresented areas, especially Africa, highlight the need for further research to guide global policies for managing and preventing obesity.

**Supplementary Information:**

The online version contains supplementary material available at 10.1007/s40200-025-01774-7.

## Introduction

Type 1 diabetes (T1D) is an autoimmune condition characterized by immune-mediated destruction of insulin-producing pancreatic beta cells [1]. For this reason, treatment must be based on insulin administration throughout the life course [2]. In recent years, there has been a rise in the burden of T1D in children and adolescents [3], posing enormous challenges to the healthcare systems globally. At the same time, obesity and overweight which predispose them to diabetes-related complications are prevalent and ubiquitous [4]. Historically, obesity was rare among individuals with T1D [5]. Today, however, the trend has changed with obesity emerging as a significant comorbidity [6], reflecting broader global trends in obesity and its impact on chronic diseases.

While there is growing evidence that obesity in T1D is still not well understood or appreciated [7], the association between T1D and obesity is complex and multifaceted. Behavioral, environmental, and biological influences in the form of physical inactivity stemming from fear of hypoglycemia [8], unhealthy and changing dietary habits [9], and genetic predisposition [10] combine in an additive manner to cause rising obesity rates among individuals with T1D. Furthermore, sociodemographic characteristics such as age, sex and disease duration have been evident to increase susceptibility to obesity [11]. Obesity is now often seen at the time of T1D diagnosis and can worsen during treatment as a result of intensive insulin regimens. Insulin therapy, an essential component of T1D management, has been associated with overweight [8, 12]. For instance, the ancillary study of the Diabetes Control and Complications Trial (DCCT) revealed that intensive insulin therapy in T1D was associated with profound weight gain, increasing body mass index (BMI), especially in the top quartile, blood pressure, and adverse lipid profiles [13].

Obesity has far-reaching clinical implications as it heightens the burden on many organ systems leading to microangiopathic complications [14], poor glycemic control [15], higher risk of insulin resistance, dyslipidemia, and cardiometabolic complications in T1D [7]. Moreover, the combination of T1D with insulin resistance, resulting from obesity, may lead to the development of double diabetes [16]. Interestingly, a Mendelian randomization study projected that a weight loss of 10% in severely obese children may prevent 22% of the cases of T1D [17], which points to a critical opportunity for prevention of the disease.

The dual burden of overweight or obesity and T1D negatively impacts effective weight management and glycemic control [8]. Despite the growing recognition of this problem, the global prevalence of obesity among children with T1D and factors leading to its development remain critically understudied. Besides, to the best of our knowledge, no review has thus far systematically synthesized the prevalence of obesity and overweight among children with T1D worldwide. Meanwhile, preliminary literature search indicated an increasing number of studies on obesity in children with T1D; however, data are still dispersed and lack comprehensive synthesis. Therefore, this systematic review and meta-analysis aimed to bridge these gaps through the establishment of the global prevalence of obesity and overweight among children and adolescents with T1D. Additionally, the study sought to analyze socio-demographic (e.g., socioeconomic status, family background), clinical (e.g., insulin use, duration of diabetes), and lifestyle factors (e.g., physical activity, dietary habits) associated with development of obesity and overweight among children with T1D. The findings are critical to develop preventive interventions for overweight or obesity and improve health outcomes for children with T1D worldwide.

## Materials and methods

This systematic review was conducted following the Preferred Reporting Items for Systematic Reviews and Meta Analyses (PRISMA) [18]. A systematic search of electronic medical databases was performed for this study to assess the current body of literature and identify any gaps in the evidence. The protocol of this review was then developed and registered and registered in PROSPERO: CRD42024591467.

### Inclusion and exclusion criteria

The inclusion criteria were: (1) observational studies (e.g., cross-sectional, cohort, and case-control design), (2) published in English Language between 2000 and 2024 among children and adolescents diagnosed with T1D (< 20 years) regardless of geographical location. The classification for overweight and obesity was done based on BMI by WHO standards, where overweight was considered in the 85th–94th percentile composition for age and sex, while obesity was considered equal to or over the 95th percentile for age and sex [19]. Moreover, the CDC BMI z-scores (standard deviation scores) was used to classify overweight as a z-score between + 1 and + 2, and obesity as a z-score greater than + 2. These criteria were consistent across the included studies. The prevalence of overweight and obesity among children with TID was the main focus of a study to be considered for this review. However, review articles, commentaries, conference abstracts, and clinical practice guidelines were excluded. Additionally, studies that had participants with T1D and T2D but did not separately report findings for the T1D participants were excluded.

### Search strategy

A systematic database search was conducted in PubMed, CINAHL, EMBASE, MEDLINE, and Web of Science, with the librarian’s support from 2000 to October 2024. The search terms included keywords and medical subject headings (MeSH) terms for “obesity”, “overweight”, “prevalence”, “associated factors”, “children and adolescents”, and “type 1 diabetes” to identify relevant studies on obesity/overweight in children with T1D. Truncation was used to include keyword variations. The Boolean operator ‘OR’ avoided exclusion by incorporating alternative but synonymous phrases, while ‘AND’ ensured that crucial words were present in the articles. The full details of the search strategy are provided in Appendix 1 (Supplementary data).

### Screening and study selection

Following database searches, the retrieved articles were uploaded into Covidence for data management and screening based on predetermined inclusion and exclusion criteria. Once duplicates were removed, the remaining articles were screened by three independent reviewers (EPO, GYO, and EE). More specifically, titles and abstracts of articles were first screened for eligibility, followed by full-text review of the potentially eligible article. Any disagreement in the screening decisions made by the three reviewers was discussed with a third reviewer, (SA), who made the final decision on the eligibility of the article.

### Data extraction

The authors developed an Excel-based data extraction form that captured information on author’s name, publication year, country, study design, participant sex, sample size, mean age, mean T1D duration, mean BMI (kg/m²), and prevalence of overweight and obesity, and associated factors. Piloting of this form was done on five randomly selected papers, and modifications were made following the pilot test. Data from each study were independently extracted by two authors (EPO and EE). Subsequently, a third author (SA) checked the data for completeness and accuracy. Any discrepancies were resolved by discussion among the two with the third author to reach a consensus.

### Quality assessment

Methodological quality of the included studies was critically appraised using the Joana Briggs Institute (JBI) checklist for cross-sectional studies and cohort studies [20]. In this regard, all the included studies were independently reviewed by two authors (EPO, EE) against the JBI criteria, and discrepancies were resolved by a third author (SA). The appraisal tool includes eight questions for cross-sectional studies and eleven questions for cohort studies with the following possible answers: “Yes”, “No”, “Unclear” and “Not applicable”. Studies that received a score of 50% or above on the JBI quality assessment indicators were deemed low risk. Full details of the quality appraisal tool are presented in Appendix 2 (Supplementary data).

### Statistical analysis

Meta-analysis was conducted in R statistical software using the meta package. The DerSimonian-Laird random-effects model was used to estimate the pooled prevalence of overweight and obesity, accounting for heterogeneity in the included studies [21]. Cochran’s Q χ² statistic and the I² test were performed to determine variability among studies. The degree of heterogeneity was determined based on the following thresholds: 0% (none), ≤ 25% (low), 25%–50% (moderate), 50%–75% (substantial), and ≥ 75% (high) [22]. Funnel plot analysis was used to investigate the presence of publication bias, which was also statistically explored using the Egger’s and Begg’s tests [23]. Subgroup analysis was conducted for gender, geographic location, and study design. In assessing factors associated with overweight and obesity among children with T1D, odds ratio was extracted from included studies, and then summarized if data were available from two or more studies.

## Results

### Search results

**Fig. 1:**
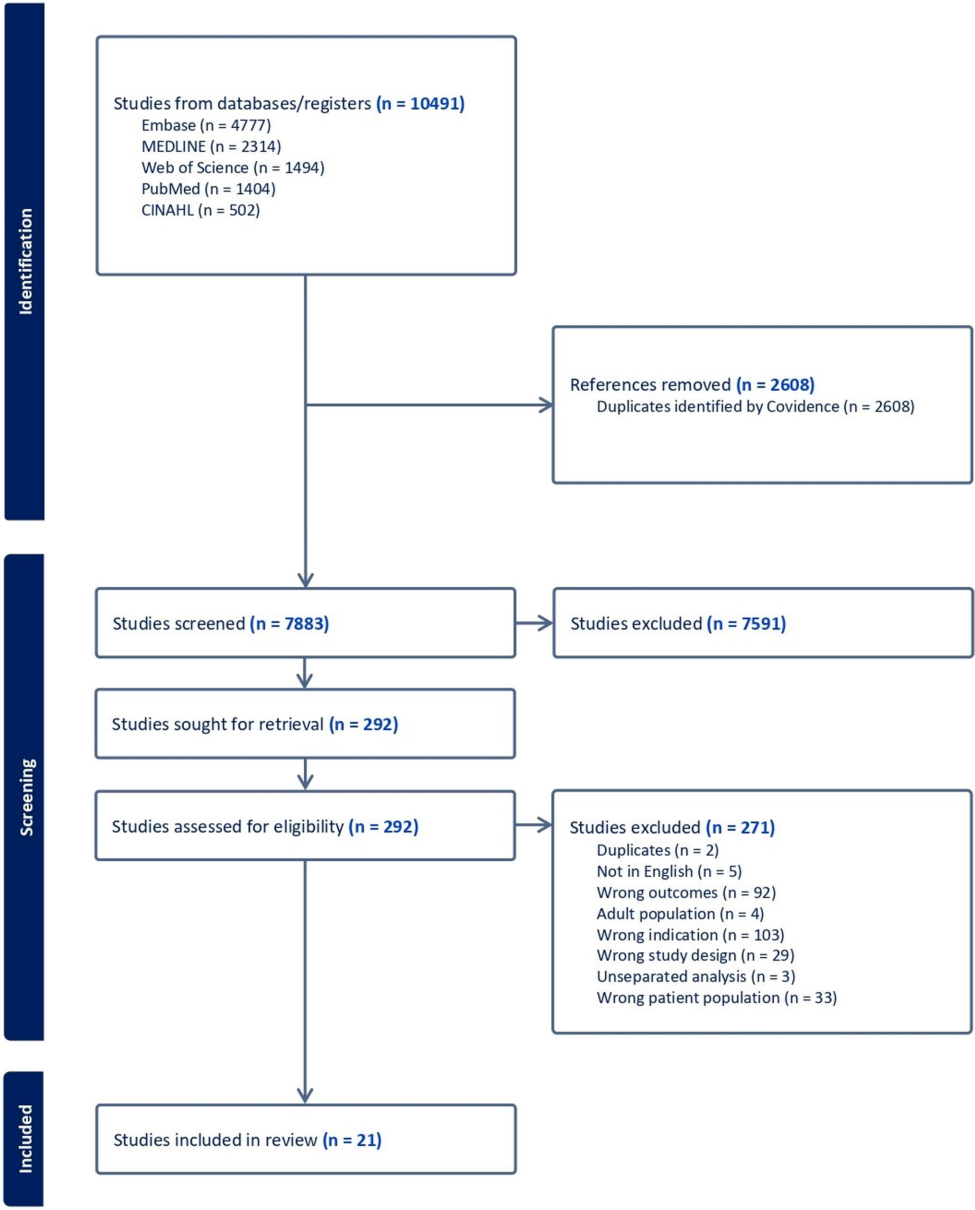
PRISMA flowchart showing the study's selection process

A total of 10,491 citations were retrieved from 5 the databases. Of these, 2,609 duplicates were removed while 7,591 citations were excluded based on the titles and their respective abstracts. Two hundred and ninety-two articles were screened for full-text review and 271 were excluded. Finally, 21 articles were selected to estimate the pooled prevalence of overweight and obesity among children and adolescents with T1D. The steps involved in the screening process are shown in detail in the PRISMA flow chart of study selection (Figure 1).

### Characteristics of included studies

Twenty-one (*n* = 21) studies were included in this systematic review and meta-analysis. These studies were published between 2008 and 2024 from a wide array of geographical regions: North America (USA, Canada) [24–30], South America (Brazil) [31, 32], Europe (Germany, Austria, Denmark, Iceland, Norway, Sweden, Netherlands, Poland) [15, 28, 33–35], Asia and Middle East (India, Malaysia, Iran, Israel) [36–39] and Australia [40]. North America led the geographical distribution of the studies, with Europe, Asia and the Middle East, South America, and Australia following in that order.

Seventeen (17) articles utilized a cross-sectional design [15, 24–27, 31, 32, 34–40] while 4 employed cohort (prospective and retrospective) study designs [15, 29, 33, 41]. The sample sizes ranged from 63 to 32,936 participants, and the total pooled sample size was 95,861 children and adolescents. The mean age of participants ranged from 9.7 to 16.4 years, with several studies reporting their medians and interquartile ranges. Duration of T1D also varied, with means from 3.9 to 8.1 years. The percentages of females across various samples were between 42.7% and 55%. Only a few studies (5) reported mean body mass index, with means ranging from 18.5 kg/m² to 21.2 kg/m². Table 1 presents the full details of the characteristics of the included studies.

**Table 1:** Characteristics of the Included Studies

First Author (Year of Publication)	Country	Study design	Sample Size	Female Sample (%)	Mean age ± SD	Mean T1D Duration ± SD	Mean BMI (kg/m2)	Overweight prevalence (%)	Obesity prevalence (%)	Obesity and overweight prevalence (%)	JBI Quality Appraisal Score
Minges 2017	USA	Cross-sectional	5529	48.2	15.4 ± 1.4	6.8 ± 4.1	NRª	22.9	13.1	36	8
Minges 2016	USA	Cross-sectional	318	55	12.3 ± 1.1	5.0 ± 3.5	21.2	24.8	14.2	39	8
Birkebaek 2018	Denmark, Iceland, Norway, and Sweden	Retrospective cohort	11,025	48	Median (IQR^b^): 13.5 (10.4; 14.4)	median: 4.3, IQR^b^ (2.2; 7.1),	NRª	18.6	18.5	37.1	5
DaCosta 2016	Brazil	Cross-sectional	195	45.6	10.6 ± 3.8	5.58 ± 3.37	NRª	30.3	9.7	40	6
Baskaran 2015	USA	Cross-sectional	136	49	12.7 ± 2.5	6.4 ± 3.2	NRª	22	10	32	5
Fröhlich-Reiterer 2014	Germany and Austria	Prospective cohort study	12,774	46.6	13.4 ± 3.9	4.7 ± 3.0	NRª	12.5	2.8	15.3	10
Tee 2022	Malaysia	Cross-sectional	63	47.6	12.4 ± 3.3	NRª	NRª	NRª	NRª	17.5	6
Oza 2022	India	Cross-sectional	355	53.5	13.7 ± 4	6.0 ± 3.9	NRª	NRª	NRª	15.5	5
Gomes 2022	Brazil	Cross-sectional	251	50.1	16.4 ± 1.9	8.1 ± 4.3	NRª	21.5	4.4	25.9	8
Phelan 2017	Australia	Retrospective cohort	3279	48	12.8 ± 3.7	5.7 ± 3.7	NRª	8	25	33	6
DuBose 2015	Germany, Austria, and the United States	Cross-sectional	32,936	48	Median: 12.6, IQR^b^ (9.5, 15.1)	Median: 4.0, (IQR^b^) (1.9, 7.0)	NRª	24	12	36	7
Manyanga 2016	Canada	Retrospective cohort	377	42.7	12 ± 3.20	NRª	NRª	15	8	23	5
Blouin 2011	Canada	Cross-sectional	70	44.3	9.7 ± 1.9	5.7 ± 2.7	18.5	15.7	5.6	21.3	5
Sands 2013	USA	Cross-sectional	243	46.4	13 ± 3	6.3 ± 3.4	NRª	23	10	33	6
Mosallanejad 2024	Iran	Cross-sectional	120	51	Median (IQR^b^): 5.5 (3–15)	5.5	NRª	10.8	2.5	13.3	6
Łuczyński 2011	Poland	Cross-sectional	500	49	Median (IQR^b^): 13.6 (10.2–15.9)	Median (IQR^b^): 4.4 (2.1–7.0)	NRª	15.6	14.6	30.2	8
Sevaliev 2019	Israel	Cross-sectional	96	50.1	14.1 ± 3.7	3.9 ± 3.1	NRª	37	11.5	48.5	6
Sandhu 2008	Canada	Cross-sectional	390	49	11.7 ± 2. 7	NRª	20.0	24.1	5.4	29.5	6
Van Vliet, et al. 2010	Nether-lands	Cross-sectional	283	48.8	Median 12.8 (IQR^b^, 9.9 to 16.0)	Median 5.3 (IQR^b^, 2.9 to 8.6)	18.5	29.3	9.2	38.5	4
Maffeis 2018	25 European countries and 12 countries outside Europe	Cross-sectional	23,026	48.5	Male: Median (IQR^b^); 14.5 (10.9 to 17.2); Female: Median (IQR^b^); 14.3 (10.9 to 17.0)	Male: Median (IQR^b^); 5.3 (3.0 to 8.6); Female: Median (IQR^b^); 5.5 (3.1 to 8.7)	Male: Median (IQR^b^); 22.1 (20.3 to 24.3); Female: Median (IQR^b^); 23.4 (21.3 to 25.9)	49.5	14.1	63.6	8
Liu 2010	USA	Cross-sectional	3,524	50	NRª	NRª	NRª	22.1	12.6	34.7	6

^a^ NR: Not Reported

^b^ IQR: Interquartile Range

BMI: Body Mass Index

JBI: Joanna Briggs Institute

### Quality of included studies

The quality scores for the included studies ranged from 4 to 8 out of 8 for cross-sectional studies and 5 to10 out of 11 for cohort studies. More than three-quarters of the studies, 76.2%, had a moderate to high quality with a score ≥ 6. The most common methodological deficiency in the reviewed studies pertained to the vague description of strategies to address confounding, which was found in many cohort studies. The outcome of the quality assessment is appended as a supplemental file [Appendix 2].

###  Prevalence of obesity

**Fig. 2:**
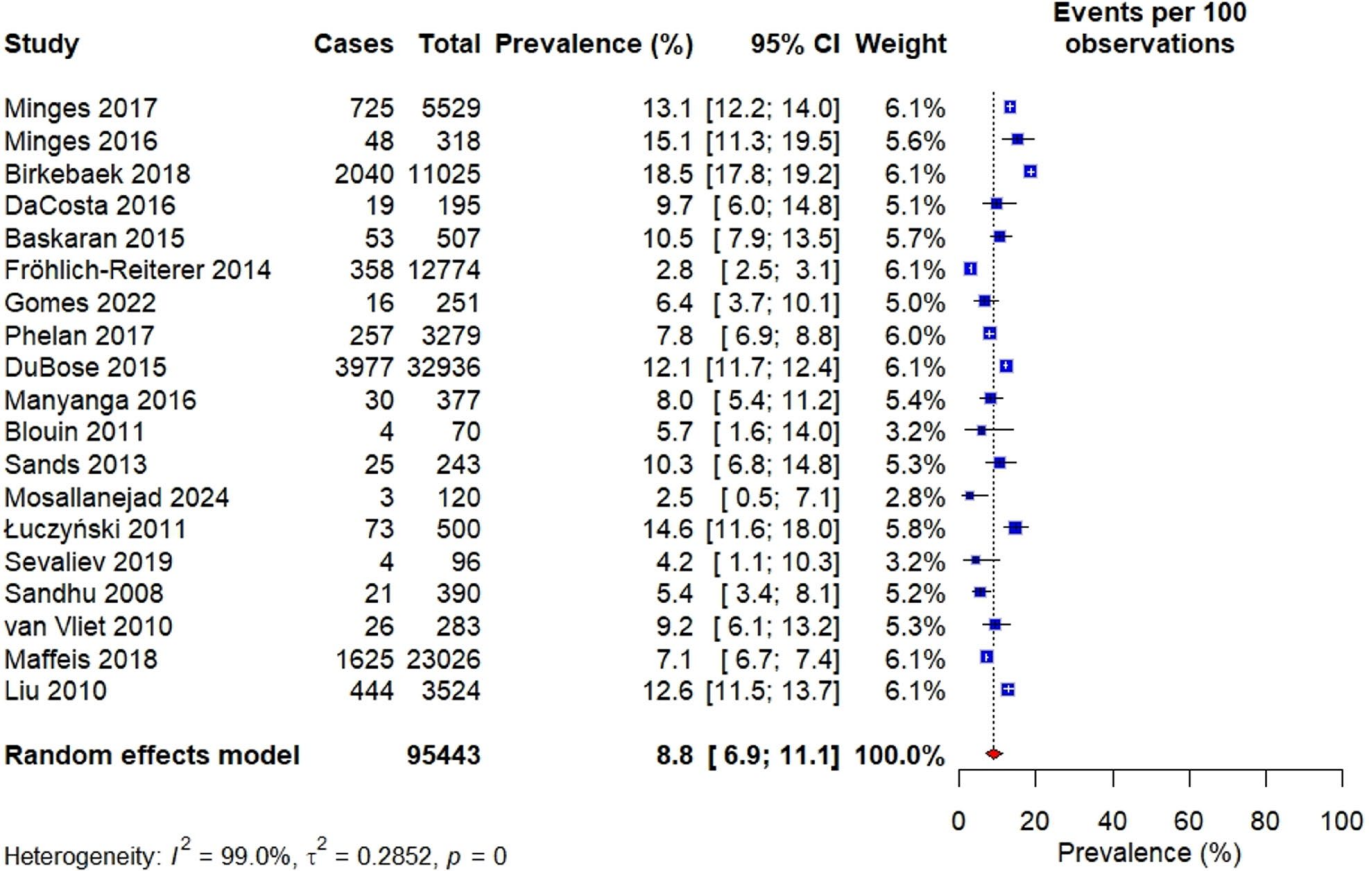
Overall prevalence obesity

Overall, the prevalence of obesity ranged from 2.5% [39] to 18.5% [33] in 19 cross-sectional and cohort studies. In random effects model, the pooled prevalence of obesity in children and adolescents with T1D was 8.8% [95% CI = 6.9–11.1], with statistically significant heterogeneity among studies (I² = 99.0%, p-value < 0.001), as shown in figure 2.

###  Prevalence of overweight

**Fig. 3:**
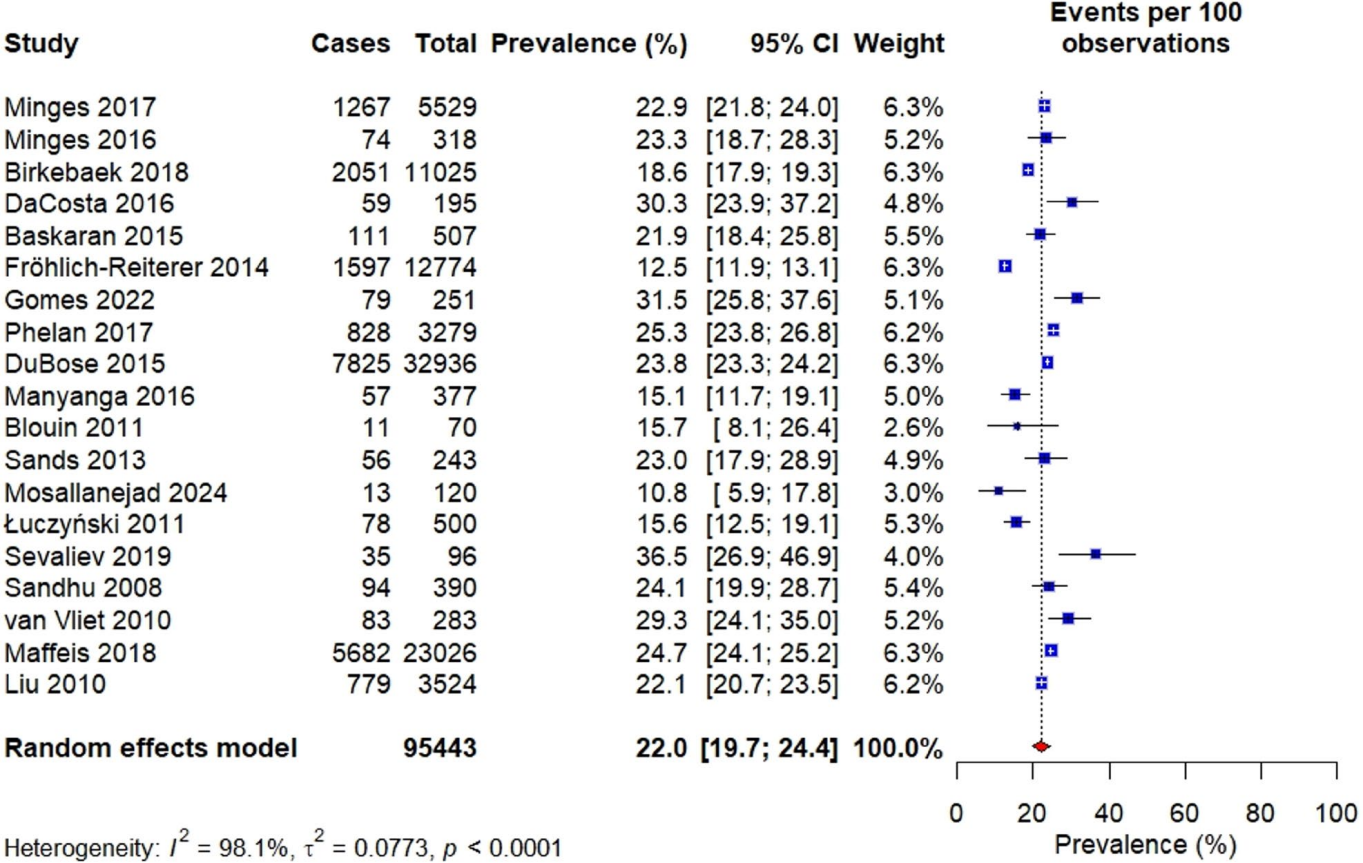
Overall prevalence of overweight

Apart from obesity, nineteen (19) studies also reported prevalence of overweight among children with T1D. The prevalence of overweight reported across different countries varied between 10.8% [39] and 49.5% [38]. In this meta-analysis, the estimated pooled prevalence of obesity among children with T1D was 22.0% [95% CI: 19.7–24.5]. However, there was a significantly high heterogeneity among the studies in the random effects model analysis (I² =98.1%, *p* < 0.001) (Figure 3).

###  Prevalence of overweight and obesity

**Fig. 4:**
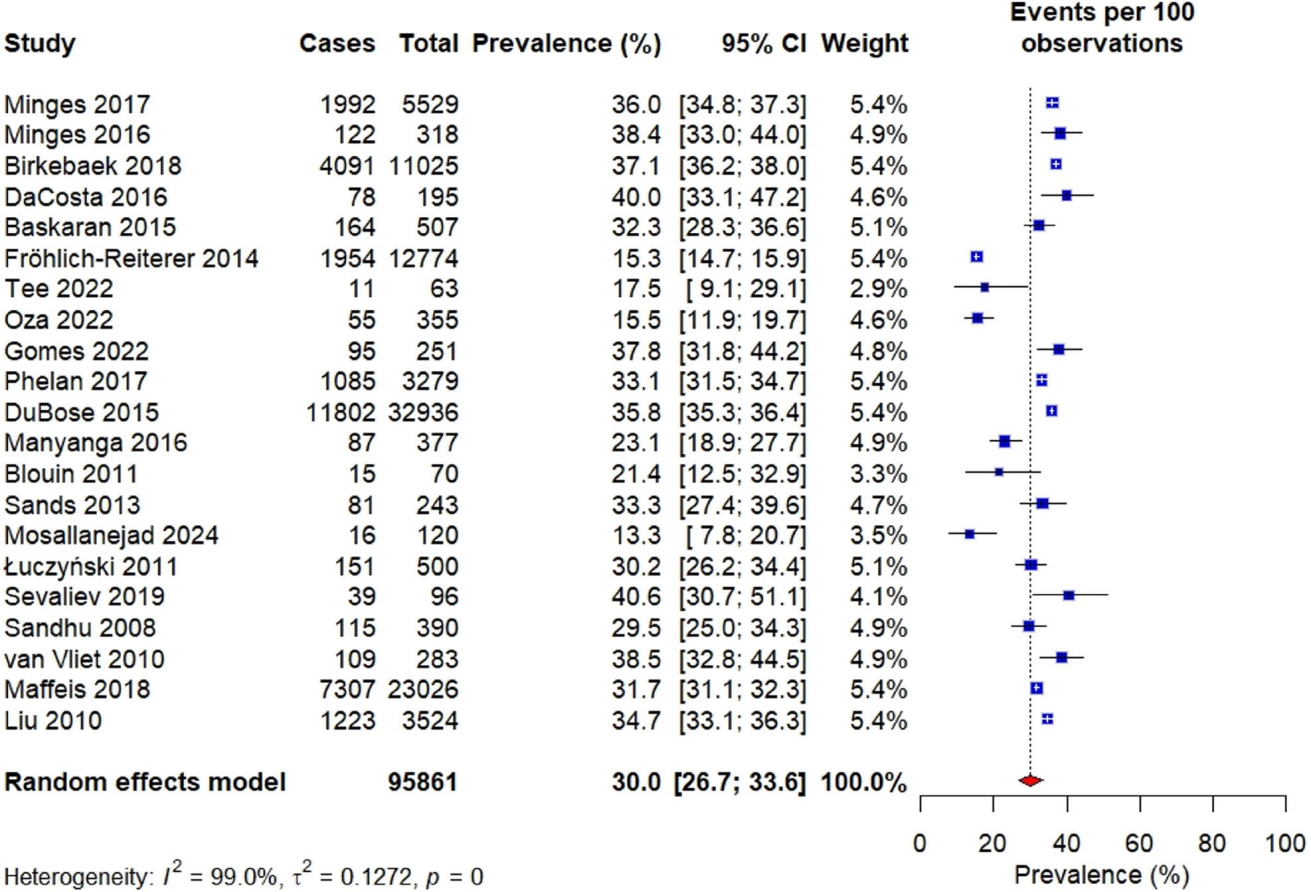
Overall prevalence of both overweight & obesity

Twenty-one (21) articles were included in the meta-analysis to estimate the overall prevalence of overweight and obesity which varied from 13.3% [39] and 63.6% [38]. For studies in which combined prevalence data were not reported but data for overweight and obesity were reported separately, the prevalence values were summed to obtain the overall prevalence of overweight and obesity. In random effects model, the pooled prevalence of overweight and obesity in children and adolescents with T1D was 30.0% [95% CI = 26.7–33.6]. There was a significant level of heterogeneity among studies (I^2^ = 99.0, P-value < 0.001) as illustrated in figure 4.

### Subgroup analysis

The included studies were categorized into five continents including North America, South America, Europe, Asia and the Middle East, and Australia. Nineteen studies reported country-specific prevalence of overweight and obesity in diverse geographical regions. However, two studies [15, 42] were excluded in the analysis because it was conducted in two different continents. In this meta-analysis, the estimated pooled prevalence of obesity and overweight among children with T1D was highest in South America 38.8% [95% CI: 34.4–43.4], followed by North America at 32.2% [95% CI: 29.6–35.0] and Europe at 29.1% [95% CI: 15.8–47.3] while Asia and the Middle East reported the lowest pool at 20.3% [95% CI: 11.1–34.2]. In Australia, the estimated prevalence derived from one single study [40] was 33.1% [95% CI: 31.5 to 34.7], which was comparable to that of North America. The subgroup analyses reduced heterogeneity from the overall analysis by 99.0%. Importantly, substantial reductions in heterogeneity were observed in subgroups such as South America and North America, with I² values of 0.0% and 82.2%, respectively, although Europe and Asia and the Middle East continued to exhibit high heterogeneity at 99.8% and 90.5%, respectively.

Moreover, of all studies included in the current meta-analysis, 7 of them [24, 25, 27, 29, 34, 35, 42] reported gender-specific data on prevalence of overweight and obesity among children with T1D. The pooled prevalence from the random effects model analysis was slightly higher in females at 17.2% [95% CI: 14.9–16.1] than in males at 15.5% [95% CI: 15.1–19.6].

The prevalence of overweight and obesity was higher in cross-sectional studies at 32.1% [95% CI: 30.1–34.1] compared to cohort studies, which reported a prevalence of 26.8% [95% CI: 17.6–38.6]. Table 2 presents the full details of the subgroup analyses.

**Table 2:** Subgroup analysis

Subgroup	No. studies	Prevalence (95% CI)	Heterogeneity ^b^ I² (*p*-value)	Subgroup difference (*p*-value)
Region/Continent				^a^ χ²=10.31 (0.035)
North America	8	32.2% [95% CI: 29.6–35.0]	82.8% (< 0.01)	
Europe	4	29.1% [95% CI: 15.8–47.3]	99.8% (< 0.01)	
South America	2	38.8% [95% CI: 34.4–43.4]	0.0% (< 0.01)	
Asia and Middle East	4	20.3% [95% CI: 11.1–34.2]	90.5% (< 0.01)	
Australia	1	33.1% [95% CI: 31.5–34.7]	––	
Gender				^a^ χ²=2.32 (0.128)
Male	7	15.5% [95% CI: 15.2–19.6%]	15.7% (< 0.01)	
Female	7	17.2% [95% CI: 14.9–16.1]	90.4% (< 0.01)	
Study design				^a^ χ²=1.58 0.208)
Cohort	4	26.8% [95% CI: 17.6–38.6]	99.8% (< 0.01)	
Cross-sectional	17	34.5% [95% CI: 30.8–38.5]	96.5% (< 0.01)	

CI: Confidence Interval

^a^ χ²: Chi-squared test for subgroup differences

^b^ I²: Heterogeneity statistic, with p-values indicating the significance of heterogeneity

### Publication bias

**Fig. 5:**
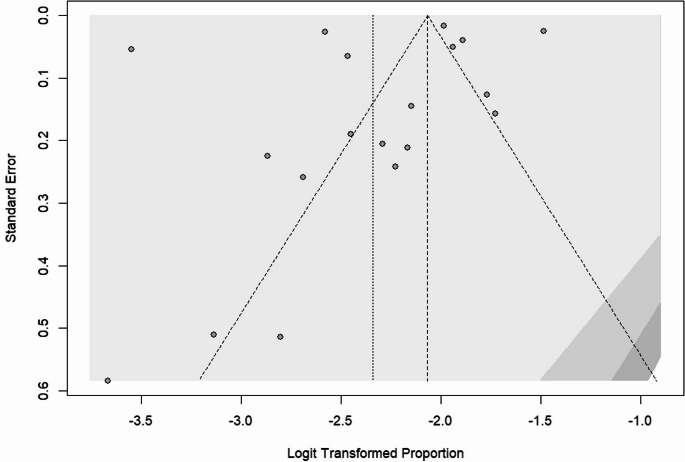
Obesity Funnel Plot

**Fig. 6:**
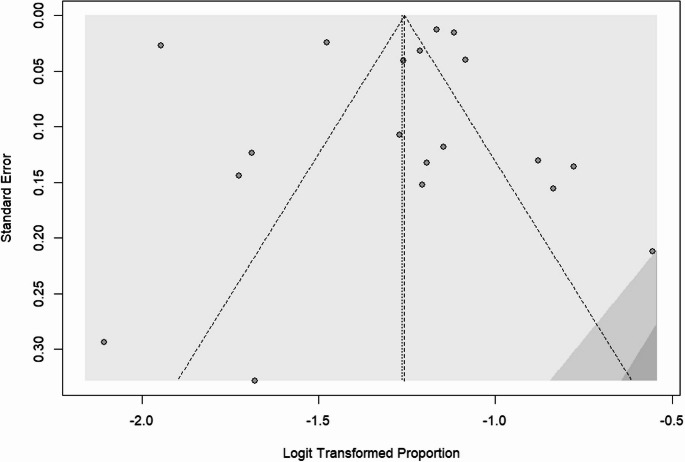
Overweight Funnel Plot

**Fig. 7:**
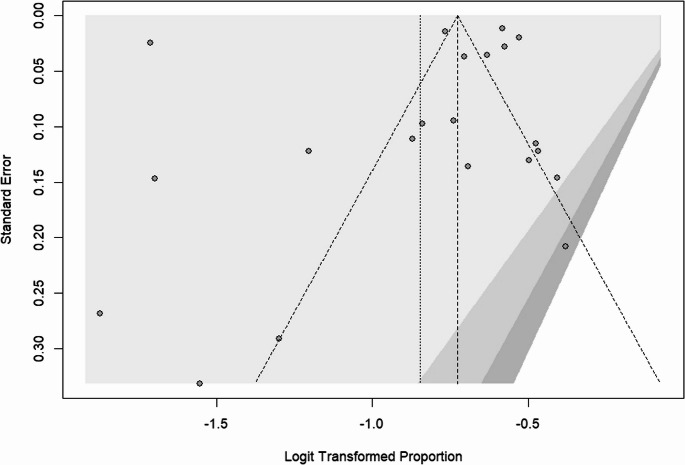
Funnel Plot - Both obesity & overweight

The publication bias was checked by a funnel plot and objectively by the Begg’s and Egger’s test. There was a publication bias among the included studies in obesity, overweight, and both obesity and overweight, as depicted by asymmetrical distribution of funnel plot tests in Figures 5, 6, and 7 respectively. However, the Egger’s test of the funnel plot did not show any significant publication bias, with p-values of 0.689 for overweight, 0.364 for obesity, and 0.511 for combined obesity and overweight. Similarly, Begg’s test showed no significant bias for overweight, with *p* = 0.689, and obesity, with *p* = 0.552, while it showed statistical significance for combined obesity and overweight, with *p* = 0.046 (Appendices).

Few studies (*n* = 6) reported factors associated with overweight and obesity in children with T1D, limiting the feasibility of a meta-analysis. Sociodemographic factors such as female gender [31, 32, 43], increasing age [24, 32], Hispanic/Latino race [24], lower household income [24], and lower parental education [43] were linked to higher overweight and obesity risk. Clinical factors included longer T1D duration [24, 25], higher HbA1c levels [24] and increased insulin doses [24, 32]. Behavioral factors such as reduced physical activity [24], lower self-monitoring of blood glucose [24], perceived stress [25], poor diabetes care activities [25], and poor quality of life [25] were significant. The results indicate the complex interaction of sociodemographic, clinical, and behavioral elements that present the risk of obesity and overweight among children with T1D. Table 3 presents the details of factors associated with overweight and obesity.

**Table 3:** Summary of the factors associated with overweight and obesity among Children with T1D

Author	Factors	Significant responds	Adjusted results OR (95% CI); *n* (%), mean ± SD or median [IQR, interquartile range]; Coefficients (B)
	DEMOGRAPHIC		
Minges 2017 DaCosta 2016 Gomes 2022	Gender	Female Female Female	1.21 (1.01–1.45) 1.81 (1.01–3.24) 57 (60.0)
Minges 2017 DaCosta 2016	Age	Yes ≥ 10	1.43 (1.34–1.52) 0.41 (0.20–0.86) B = − 0.0616 [− 0.0881; −0.0350] B = − 0.0733 [− 0.1044; −0.0423] 16.9 ± 1.8
Minges 2017	Race	Hispanic/Latino	1.33 (1.04–1.71
Minges 2017	Annual Household income	<$35,000 $35,000–$74,999	2.24 (1.39–3.60) 1.87 (1.21–2.88)
Minges 2017 Minges 2016	Parental educational attainment	High school Some college Bachelor’s or associate degree Associate degree or some college High school diploma or less	1.64 (1.19–2.26) 1.39 (1.02–1.89) 1.29 (1.00–1.67) 4.38 (1.54–12.50) 1.13 (1.03–1.23)
Minges 2017 Minges 2016 Birkebaek 2018 Gomes 2022	Duration of T1D	Yes Yes Categorical, Yes, ALL + VE Yes	0.97 (0.95–1.00) 1.13 (1.03–1.23) B = 0.0768 [0.0591; 0.0944 8.9 ± 4.4
Gomes 2022	Level of care, tertiary	Yes	63(66.3)
Gomes 2022	Geographic region	Yes	< 0.001
Gomes 2022	Family history of Type 2 Diabetes	Yes	49(53.3
	CLINICAL		
Minges 2017 Nordic 2018	HbA1c	Yes > 58.1 mmol/mol	0.81 (0.76–0.87) B = − 0.0409 [− 0.0538; −0.0280
Minges 2017	Bolus/short-acting insulin	Yes	1.01 (1.01–1.02)
Minges 2017	Basal/long-acting insulin	Yes	1.06 (1.05–1.07)
Birkebaek 2018 Gomes 2022 Gomes 2022	Insulin dose	> 0.61–0.80 IU/kg/d Insulin dose (U/kg/day) Insulin dose, total (U/day)	B = 0.0382 [0.0170; 0.0594] 0.95 ± 0.4 65.67 ± 25.2
Minges 2017	Severe hypoglycemia	Yes	1.96 (1.06–3.64)
Minges 2017	Self-rated health	Very good Good Fair/poor	1.75 (1.28–2.39) 3.80 (2.77–5.20) 5.77 (3.85–8.65)
DaCosta 2016	Daily dose of insulin/kg	Yes	2.12 (1.13–3.97)
Gomes 2022	Total Cholesterol (mg/dL)	Yes	197.1 ± 57.2
Gomes 2022	LDL-cholesterol (mg/dL)	Yes	120.2 ± 39.4
Gomes 2022	LDL-cholesterol ≥ 100 mg/dl, n(%)	Yes	141.3 ± 51.3
Sevaliev 2019	HDL, mg/dL	Yes	50 ± 13.8
Gomes 2022	GFR, mL/min/1.73m2	Yes	106.8 ± 23.4
Gomes 2022	Diastolic blood pressure	Yes	73.9 ± 8.4
Sevaliev 2019 Gomes 2022	Systolic blood pressure	Yes Yes	120.5 ± 14.2 118.0 ± 10.3
	BEHAVIORAL		
Minges 2017	Self-monitoring blood glucose	Yes	0.94 (0.89–0.98)
Minges 2017	Insulin to carbohydrate ratio	Yes	0.94 (0.92–0.96)
Minges 2017	Physical activity	Active	0.70 (0.49–0.99)
Minges 2016	Perceived stress	Yes	1.07 (1.02–1.11)
Minges 2016	diabetes care activities	Yes	0.94 (0.89–0.99)
Minges 2016	general quality of life	Yes	0.97 (0.94–0.99)

## Discussion

This meta-analysis examined the global overweight and obesity prevalence among children with T1D across five continents in studies published from 2000 to 2024. To the best of our knowledge, this study is the first systematic review and meta-analysis that comprehensively evaluated the prevalence of overweight and obesity in this population worldwide.

In this study, the overall pooled prevalence of obesity and overweight among children and adolescents was 30.0%. This prevalence delineates the growing burden of weight-related problems in this population, which may exacerbate the challenges of managing glycemic control and increase the risk of diabetes-related complications. In addition, the pooled prevalence of obesity alone was 8.8% while overweight prevalence was higher at 22.0%. Evidence from a meta-analysis that was conducted among the general children and adolescents population supports these findings which also depicted that 1 in 5 children or adolescents had excess weight [44]. Besides, several studies attributed this increase in weight to lifestyle changes, such as energy-dense, ultra-processed foods with high fats and carbohydrates but low in essential nutrients, combined with reduced physical activity due to sedentary activities [44, 45]. Diet quality is critical for child development, and poor dietary habits are a modifiable risk factor for obesity [45, 46]. Beyond increasing cardiovascular risk, obesity and insulin resistance can alter the clinical presentation and natural history of T1D [47], which in turn can affect subsequent treatment. This underscores the need to address obesity and overweight in T1D early to improve patient outcomes and prevent further complications.

Furthermore, this meta-analysis highlighted considerable variations in the prevalence of overweight and obesity among children with T1D across the five continents. The estimated pooled prevalence was highest in South America (38.8%), followed by North America (32.2%) and Europe at 29.1%, while Asia and the Middle East reported the lowest pool at 20.3%. This geographical disparity in prevalence could be attributed to factors such as dietary practices, way of life, and health care accessibility, leading to overweight or obesity in T1D. It is therefore imperative to implement country or region-specific interventions in attempts to deal with rising obesity and related complications while managing T1D.

Moreover, the pooled prevalence of overweight and obesity was slightly higher in females (17.2%) compared to males (15.5%). This discrepancy, though modest, could be explained by several factors including body composition, hormone biology, and susceptibility to certain social, patterns of weight gain, ethnic, genetic, and environmental factors that explain gender differences [48]. Similarly, Ingberg et al. (2004) argued that puberty increases the BMI standard deviation score, particularly in female teenagers with T1D [49].

The prevalence of overweight and obesity in cross-sectional and cohort studies was 32.1% and 26.8% respectively. This difference may be a result of the intrinsic differences in study design, whereby cross-sectional studies provide a snapshot of weight status at one point in time, while cohort studies track changes over time and may capture temporal fluctuations in weight.

The paucity of data on the factors that contribute to overweight and obesity in children T1D underscores the need for further studies. Sociodemographic factors such as female sex, advancing age, and socioeconomic disparities associated with an increased risk for overweight and obesity in children with T1D are consistent with the results of Maffeis et al. (2012) who reported similar trends in their cross-sectional study of pediatric diabetes patients [50]. Moreover, clinical aspects including higher levels of HbA1c, dyslipidemia, and behavioral factors such as reduced physical activity and lower self-monitoring of blood glucose identified as key contributors to obesity in this population aligns with the results of a further study by Maffeis et al. (2018) [42]. Such findings indicate that the risk of obesity in children with T1D is complex and multifaceted, stemming from an interplay between sociodemographic, clinical, and behavioral factors.

The study has several implications. First, the high prevalence of overweight and obesity among children with T1D calls for targeted interventions aimed at weight management in this population. National and international health policymakers should tackle this by implementing measures such as encouraging physical activity, promoting healthy eating habits, and incorporating weight management into standard diabetic care. Additionally, in practice, healthcare providers should be aware of the unique challenges that children with TID face when attempting to integrate insulin treatment with weight-control measures.

### Strengths and limitations

This study has several limitations. First, there was high heterogeneity between studies due to diversity in regions that may affect the certainty of our estimates. The heterogeneity of findings was attributed to differences in the study design, sample size, and regional setting. Second, most studies were from North America, South America, Europe, and Asia, with few from other regions. Only one of the studies involved Australia, and none were conducted in Africa. This might limit the representation to the world, as data was not available from all continents. Third, the search was limited to papers published in the English language, possibly excluding relevant studies published in other languages. This limitation also points toward caution while interpreting the results. Despite these limitations, the major strength of this review is its novelty, as it will provide a benchmark for future studies to be conducted on obesity in children with T1D in underrepresented regions, particularly Africa. To the best of our knowledge, this review represents the first systematic review and meta-analysis to summarize the global prevalence and factors associated with obesity in children with T1D.

## Conclusion

This meta-analysis revealed a high burden of overweight and obesity among children and adolescents with T1D globally, with significant variations by geographical region, gender, and study design. These findings further support the need for targeted interventions and policies that address the dual burden of diabetes and weight-related comorbidities in this group. Future research should focus on longitudinal studies to understand the causal pathways linking T1D to overweight and obesity and to define the effective strategies of prevention and management.

## Supplementary Information

Below is the link to the electronic supplementary material.

Supplementary Material 1 (DOCX 28.2 KB)

Supplementary Material 2 (DOCX 22.4 KB)

## Data Availability

All the data are available in the article and its supplemental files.

## Abbreviations

BMI: Body mass index
CDC: Centers for Disease Control and Prevention
JBI: Joanna Briggs Institute
T1D: Type–1 Diabetes
WHO: World Health Organization
