# Supplementary material for: Global prevalence and factors associated with overweight and obesity in children and adolescents with type 1 diabetes: a systematic review and meta-analysis
Source: J Diabetes Metab Disord. 2025 Nov 4;24(2):257. doi: 10.1007/s40200-025-01774-7 (PMC12586262; doi:10.1007/s40200-025-01774-7)
Supplement: Supplementary file 1 — Supplementary Material 1 (DOCX 28.2 KB) [file 40200_2025_1774_MOESM1_ESM.docx]

**APPENDIX 1. SEARCH STRATEGY USED FOR THE ELECTRONIC DATABASES (10.5.2024)**

1. **PubMed database search: 1404 results**

| **#** | **Concept** | **Searches** | **Results** |
| --- | --- | --- | --- |
|  | Obesity | **("Obesity"[Mesh] OR "Pediatric Obesity"[Mesh] OR "Body Mass Index"[Mesh] OR "Overweight"[Mesh] OR "Body Weight"[Mesh]) OR ("severe obesity"[Title/Abstract] OR "extreme obesity"[Title/Abstract] OR "body fat"[Title/Abstract] OR "adiposity"[Title/Abstract] OR "fat accumulation"[Title/Abstract] OR "obese"[Title/Abstract] OR "obese patien*"[Title/Abstract] OR "overweightness"[Title/Abstract] OR "BMI"[Title/Abstract])** | [797,058](https://pubmed.ncbi.nlm.nih.gov/?term=%28%22Obesity%22%5BMesh%5D+OR+%22Pediatric+Obesity%22%5BMesh%5D+OR+%22Body+Mass+Index%22%5BMesh%5D+OR+%22Overweight%22%5BMesh%5D+OR+%22Body+Weight%22%5BMesh%5D%29+OR+%28%22severe+obesity%22%5BTitle%2FAbstract%5D+OR+%22extreme+obesity%22%5BTitle%2FAbstract%5D+OR+%22body+fat%22%5BTitle%2FAbstract%5D+OR+%22adiposity%22%5BTitle%2FAbstract%5D+OR+%22fat+accumulation%22%5BTitle%2FAbstract%5D+OR+%22obese%22%5BTitle%2FAbstract%5D+OR+%22obese+patien%2A%22%5BTitle%2FAbstract%5D+OR+%22overweightness%22%5BTitle%2FAbstract%5D+OR+%22BMI%22%5BTitle%2FAbstract%5D%29&size=200&sort=relevance) |
|  | Prevalence/  associated factors | **(("Prevalence"[Mesh]) OR ("Risk Factors"[Mesh]) OR ("Epidemiology"[Mesh]) OR ("Association"[Mesh])) OR ("Prevalence"[Title/Abstract] OR "prevalen*"[Title/Abstract] OR "risk factors"[Title/Abstract] OR "associate"[Title/Abstract] OR "precipitating factors"[Title/Abstract] OR "precipitating factors"[Title/Abstract] OR "precipitat*"[Title/Abstract])** | 2,504,946 |
|  | Children/  Adolescents/ | **(("Child"[Mesh]) OR ("Adolescent"[Mesh])) OR (child[Title/Abstract] OR children[Title/Abstract] OR childhood[Title/Abstract] OR pediatric[Title/Abstract] OR "school-aged child"[Title/Abstract] OR "school-age children"[Title/Abstract] OR adolescent[Title/Abstract] OR adolescents[Title/Abstract] OR teenager[Title/Abstract] OR teenagers[Title/Abstract] OR teen[Title/Abstract] OR Children[Title/Abstract] OR preadolescen*[Title/Abstract] OR p#ediatric[Title/Abstract])** | 4,096,620 |
|  | Diabetes (TIDM) | **(Diabetes Mellitus, Type 1 OR "Diabetes Mellitus, Type 1"[mesh] OR diabetes mellitus type 1 OR "diabetes mellitus type 1"[tiab] OR diabetes mellitus, type I OR "diabetes mellitus, type I"[tiab] OR diabetes type 1 OR "diabetes type 1"[tiab] OR diabetes type I OR "diabetes type I"[tiab] OR DM 1 OR "DM 1"[tiab] OR IDDM OR "IDDM"[tiab] OR IDDM1 OR "IDDM1"[tiab] OR insulin dependent diabetes OR "insulin dependent diabetes"[tiab] OR insulin-dependent diabetes mellitus OR "insulin-dependent diabetes mellitus"[tiab] OR T1DM OR "T1DM"[tiab] OR type 1 diabetes OR "type 1 diabetes"[tiab] OR type 1 diabetes mellitus OR "type 1 diabetes mellitus"[tiab] OR type I diabetes OR "type I diabetes"[tiab] OR type I diabetes mellitus OR "type I diabetes mellitus"[tiab] OR insulin dependent diabetes mellitus OR "insulin dependent diabetes mellitus"[tiab])** | 188,313 |
|  | #1 AND #2 AND #3 AND #4 | 1 AND #2 AND #3 AND #4 | 1,625 |
|  | **Filters: Years** | **2000 – 10/19/2024** | 1,404 |

1. **CINAHL database search: 2,206 results**

| **#** | **Concept** | **Searches** | **Results** |
| --- | --- | --- | --- |
|  | Obesity | (MH "Obesity") OR (MH "Pediatric Obesity") OR "obesity" OR "overweight" OR "fat" OR "obese" OR "unhealthy weight" OR "high bmi" OR "high body mass index" | 223,929 |
|  | Prevalence/  associated factors | (MH "Prevalence") OR (MH "Incidence") OR (MH "Risk Factors") OR  "prevalence" OR "incidence" OR "epidemiology" OR "frequency" OR "occurrence"  OR "risk factors" OR "contributing factors" OR "predisposing factors" OR "predictor" OR "cause" OR "vulnerability factors" | 1,512,297 |
|  | Children/  Adolescents/ | (MH "Child+") OR (MH "Adolescence") OR AB("adolescen*") OR TI("adolescen*") OR AB("Kids") OR TI("Kids") OR AB("children") OR TI("children") | 1,280,505 |
|  | Diabetes (TIDM) | ((MH "Diabetes Mellitus, Type 1+") OR AB("diabetes mellitus type 1") OR TI("diabetes mellitus type 1") OR AB("type 1 diabetes") OR TI("type 1 diabetes") OR AB("diabetes type 1") OR TI("diabetes type 1") OR AB("diabetes type I") OR TI("diabetes type I") OR AB("type 1 diabetes mellitus") OR TI("type 1 diabetes mellitus") OR AB("DM 1") OR TI("DM 1") OR AB("T1DM") OR TI("T1DM") OR AB("IDDM") OR TI("IDDM") OR AB("IDDM1") OR TI("IDDM1") OR AB("insulin dependent diabetes") OR TI("insulin dependent diabetes") OR AB("insulin-dependent diabetes mellitus") OR TI("insulin-dependent diabetes mellitus") OR AB("type I diabetes") OR TI("type I diabetes") OR AB("type I diabetes mellitus") OR TI("type I diabetes mellitus") OR AB("insulin dependent diabetes mellitus") OR TI("insulin dependent diabetes mellitus")) | 36,972 |
|  | #1 AND #2 AND #3 AND #4 | S1 AND S2 AND S3 AND S4 | 538 |
|  | **Filters: Years** | **2000 – 10/14/2024** | **502** |

1. **EMBASE database search: 4,341 results**

| **#** | **Concept** | **Searches** | **Results** |
| --- | --- | --- | --- |
|  | Obesity | 'obesity'/exp OR 'body weight, excess':ti,ab OR overweight OR 'body mass index':ti,ab | 1,096,601 |
|  | Prevalence/  associated factors | 'prevalence'/exp OR 'risk factor'/exp OR 'risk factor' OR 'predictors':ti,ab OR 'disease predisposition':ti,ab | 2,886,438 |
|  | Children/  Adolescents/ | 'child'/exp OR 'children':ti,ab OR 'adolescent'/exp OR 'teenager':ti,ab | 4,845,496 |
|  | Diabetes (TIDM) | 'insulin dependent diabetes mellitus'/exp OR 'insulin dependent diabetes mellitus' OR 'diabetes mellitus type 1':ti,ab OR 'type 1 diabetes':ti,ab OR 'diabetes type 1':ti,ab OR 'diabetes type i':ti,ab OR 'type 1 diabetes mellitus':ti,ab OR 'dm 1':ti,ab OR 't1dm':ti,ab OR 'iddm':ti,ab OR 'iddm1':ti,ab OR 'insulin dependent diabetes':ti,ab OR 'insulin-dependent diabetes mellitus':ti,ab OR 'type i diabetes':ti,ab OR 'type i diabetes mellitus':ti,ab | 518,632 |
|  | #1 AND #2 AND #3 AND #4 | #1 AND #2 AND #3 AND #4 | 4895 |
|  | **Filters: Years**  **2000 – 10/14/2024** | #5 AND (2000:py OR 2001:py OR 2002:py OR 2003:py OR 2004:py OR 2005:py OR 2006:py OR 2007:py OR 2008:py OR 2009:py OR 2010:py OR 2011:py OR 2012:py OR 2013:py OR 2014:py OR 2015:py OR 2016:py OR 2017:py OR 2018:py OR 2019:py OR 2020:py OR 2021:py OR 2022:py OR 2023:py OR 2024:py) | 4,777 |

1. **MEDLINE through OVID database search: 2313** **results**

| **#** | **Concept** | **Searches** | **Results** |
| --- | --- | --- | --- |
|  | Obesity | exp obesity/ OR body weight, excess.mp. OR overweight.mp. OR body mass index.mp. | 541381 |
|  | Prevalence/  associated factors | exp prevalence/ OR exp risk factor/ OR predictors.mp. OR exp causality/ OR exp precipitating factors/ OR predisposing factors.mp. | 1564913 |
|  | Children/  Adolescents/ | exp child/ OR children.mp. OR exp adolescent/ OR teenager.mp. | 3807185 |
|  | Diabetes (TIDM) | exp insulin dependent diabetes mellitus/ or exp non insulin dependent diabetes mellitus/ or diabetes mellitus type 1.mp. or IDDM.mp. or T1DM.mp. or type 1 diabetes.mp. | 273521 |
|  | #1 AND #2 AND #3 AND #4 | 1 and 2 and 3 and 4 | 2342 |
|  | **Filters: Years**  **2000 – 10/14/2024** | limit 5 to yr="2000 - 2024" | 2313 |

1. **WEB OF SCIENCE database search: results**

| **#** | **Concept** | **Searches** | **Results** |
| --- | --- | --- | --- |
|  | Obesity | TS=("Obesity" OR "Pediatric Obesity" OR "Body Mass Index" OR "Overweight" OR "Body Weight" OR "severe obesity" OR "extreme obesity" OR "body fat" OR "adiposity" OR "fat accumulation" OR "obese" OR "obese patien*" OR "overweightness") | 1,012,764 |
|  | Prevalence/  associated factors | TS=(**"Prevalence" OR "Risk Factors" OR "Epidemiology" OR "Association" OR "Prevalence" OR "prevalen*" OR "risk factors" OR "associate" OR "precipitating factors" OR "precipitating factors" OR "precipitat*")** | 5,006,792 |
|  | Children/  Adolescents/ | TS=(**"Child" OR "Adolescent" OR “child” OR “children” OR “childhood” OR “pediatric” OR "school-aged child" OR "school-age children" OR “adolescen*” OR preadolescen* OR “p#ediatric”)** | 2,787,214 |
|  | Diabetes (TIDM) | TS=("Diabetes Mellitus, Type 1" OR "diabetes mellitus type 1" OR "type 1 diabetes" OR "diabetes type 1" OR "diabetes type I" OR "type 1 diabetes mellitus" OR "DM 1" OR "T1DM" OR "IDDM" OR "IDDM1" OR "insulin dependent diabetes" OR "insulin-dependent diabetes mellitus" OR "type I diabetes" OR "type I diabetes mellitus" OR "insulin dependent diabetes mellitus") | 103,714 |
|  | #1 AND #2 AND #3 AND #4 | TS=("Obesity" OR "Pediatric Obesity" OR "Body Mass Index" OR "Overweight" OR "Body Weight" OR "severe obesity" OR "extreme obesity" OR "body fat" OR "adiposity" OR "fat accumulation" OR "obese" OR "obese patien*" OR "overweightness") AND TS=("Prevalence" OR "Risk Factors" OR "Epidemiology" OR "Association" OR "Prevalence" OR "prevalen*" OR "risk factors" OR "associate" OR "precipitating factors" OR "precipitating factors" OR "precipitat*") AND TS=("Child" OR "Adolescent" OR “child” OR “children” OR “childhood” OR “pediatric” OR "school-aged child" OR "school-age children" OR “adolescen*” OR preadolescen* OR “p#ediatric”) AND TS=("Diabetes Mellitus, Type 1" OR "diabetes mellitus type 1" OR "type 1 diabetes" OR "diabetes type 1" OR "diabetes type I" OR "type 1 diabetes mellitus" OR "DM 1" OR "T1DM" OR "IDDM" OR "IDDM1" OR "insulin dependent diabetes" OR "insulin-dependent diabetes mellitus" OR "type I diabetes" OR "type I diabetes mellitus" OR "insulin dependent diabetes mellitus") | 1,596 |
|  | **Filters: Years** | **2010 – 10/14/2024** | 1,494 |
