# Supplementary material for: Global prevalence and factors associated with overweight and obesity in children and adolescents with type 1 diabetes: a systematic review and meta-analysis
Source: J Diabetes Metab Disord. 2025 Nov 4;24(2):257. doi: 10.1007/s40200-025-01774-7 (PMC12586262; doi:10.1007/s40200-025-01774-7)
Supplement: Supplementary file 2 — Supplementary Material 2 (DOCX 22.4 KB) [file 40200_2025_1774_MOESM2_ESM.docx]

**Appendix 2**

**JBI Checklist for Analytical Cross-Sectional Studies**

| **First Author (Year)** | **Were the criteria for inclusion in the sample clearly defined?** | **Were the study subjects and the setting described in detail?** | **Was the exposure measured in a valid and reliable way?** | **Were objective, standard criteria used for measurement of the condition?** | **Were confounding factors identified?** | **Were strategies to deal with confounding factors stated?** | **Were the outcomes measured in a valid and reliable way?** | **Was appropriate statistical analysis used?** | **Total score** |
| --- | --- | --- | --- | --- | --- | --- | --- | --- | --- |
| Minges 2017 | Yes | Yes | Yes | Yes | Yes | Yes | Yes | Yes | 8 |
| Minges 2016 | Yes | Yes | Yes | Yes | Yes | Yes | Yes | Yes | 8 |
| DaCosta 2016 | Yes | Yes | Yes | Yes | No | No | Yes | Yes | 6 |
| Baskaran 2015 | Yes | Yes | Yes | Yes | No | No | Unclear | Yes | 5 |
| Tee 2022 | Yes | Yes | Yes | Yes | No | No | Yes | Yes | 6 |
| Oza 2022 | Yes | Yes | Unclear | Yes | No | No | Yes | Yes | 5 |
| Gomes 2022 | Yes | Yes | Yes | Yes | Yes | Yes | Yes | Yes | 8 |
| Sands 2013 | Yes | Yes | Yes | Yes | No | No | Yes | Yes | 6 |
| Mosallanejad 2024 | Yes | Yes | Yes | Yes | No | No | Yes | Yes | 6 |
| Łuczyński 2011 | Yes | Yes | Yes | Yes | Yes | Yes | Yes | Yes | 8 |
| Sevaliev 2019 | Yes | Yes | Yes | Yes | Unclear | No | Yes | Yes | 6 |
| Sandhu 2008 | Yes | Yes | Yes | Yes | No | No | Yes | Yes | 6 |
| van Vliet 2010 | Yes | Yes | Yes | No | No | No | No | Yes | 4 |
| Phelan 2017 | Yes | Yes | Yes | Yes | Unclear | Unclear | Yes | Yes | 6 |
| Blouin 2011 | Yes | Yes | Yes | Yes | No | No | Yes | Yes | 6 |
| Maffeis 2018 | Yes | Yes | Yes | Yes | Yes | Yes | Yes | Yes | 8 |
| Liu 2010 | Yes | Yes | Yes | Yes | Unclear | No | Yes | Yes | 6 |

**JBI Checklist for Cohort Studies**

| **First Author (Year)** | **Were the two groups similar and recruited from the same population?** | **Were the exposures measured similarly to assign people to both exposed and unexposed groups?** | **Was the exposure measured in a valid and reliable way?** | **Were confounding factors identified?** | **Were strategies to deal with confounding factors stated?** | **Were the groups/ participants free of the outcome at the start of the study (or at the moment of exposure)?** | **Were the outcomes measured in a valid and reliable way?** | **Was the follow up time reported and sufficient to be long enough for outcomes to occur?** | **Was follow up complete, and if not, were the reasons to loss to follow up described and explored?** | **Were strategies to address incomplete follow up utilized?** | **Was appropriate statistical analysis used?** | **Total score** |
| --- | --- | --- | --- | --- | --- | --- | --- | --- | --- | --- | --- | --- |
| Birkebaek 2018 | Yes | Yes | Yes | Yes | Yes | Yes | Yes | Yes | Unclear | Unclear | Yes | 5 |
| Fröhlich-Reiterer 2014 | Yes | Yes | Yes | Yes | Yes | Yes | Yes | Yes | Yes | Unclear | Yes | 10 |
| Manyanga 2016 | Yes | Not applicable | Yes | No | No | Yes | Yes | Not applicable | Not applicable | Not applicable | Yes | 5 |
| DuBose 2015 | Unclear | Yes | Yes | Unclear | No | Yes | Yes | Yes | Yes | Not applicable | Yes | **7** |
